# Mindful Coping Power Effects on Children’s Autonomic Nervous System Functioning and Long-Term Behavioral Outcomes

**DOI:** 10.3390/jcm12113621

**Published:** 2023-05-23

**Authors:** Caroline L. Boxmeyer, Catanya G. Stager, Shari Miller, John E. Lochman, Devon E. Romero, Nicole P. Powell, Chuong Bui, Lixin Qu

**Affiliations:** 1 Department of Psychiatry and Behavioral Medicine, College of Community Health Sciences, The University of Alabama, Tuscaloosa, AL 35487, USA; 2 Center for Youth Development and Intervention, The University of Alabama, Tuscaloosa, AL 35487, USA; jlochman@ua.edu (J.E.L.); npowell@ua.edu (N.P.P.); cnbui@ua.edu (C.B.); 3 Division of Preventive Medicine, Heersink School of Medicine, The University of Alabama at Birmingham, Birmingham, AL 35294, USA; cgstager@uabmc.edu; 4 Frank Porter Graham Child Development Institute, University of North Carolina, Chapel Hill, NC 27516, USA; sharipeace@gmail.com; 5 Department of Psychology, College of Arts and Sciences, The University of Alabam, Tuscaloosa, AL 35487, USA; 6 Department of Counseling, University of Texas at San Antonio, San Antonio, TX 78249, USA; devon.romero@utsa.edu; 7 Alabama Life Research Institute, The University of Alabama, Tuscaloosa, AL 35487, USA; 8 Carolina Population Center, University of North Carolina, Chapel Hill, NC 27516, USA; lxqu@email.unc.edu

**Keywords:** Mindful Coping Power, reactive aggression, autonomic nervous system, respiratory sinus arrhythmia, skin conductance, polyvagal theory, mindfulness, inhibitory control, self-regulation, prevention

## Abstract

Mindful Coping Power (MCP) was developed to enhance the effects of the Coping Power (CP) preventive intervention on children’s reactive aggression by integrating mindfulness training into CP. In prior pre–post analyses in a randomized trial of 102 children, MCP improved children’s self-reported anger modulation, self-regulation, and embodied awareness relative to CP but had fewer comparative effects on parent- and teacher-reported observable behavioral outcomes, including reactive aggression. It was hypothesized that MCP-produced improvements in children’s internal awareness and self-regulation, if maintained or strengthened over time with ongoing mindfulness practice, would yield improvements in children’s observable prosocial and reactive aggressive behavior at later time points. To appraise this hypothesis, the current study examined teacher-reported child behavioral outcomes at a one-year follow-up. In the current subsample of 80 children with one-year follow-up data, MCP produced a significant improvement in children’s social skills and a statistical trend for a reduction in reactive aggression compared with CP. Further, MCP produced improvements in children’s autonomic nervous system functioning compared with CP from pre- to post-intervention, with a significant effect on children’s skin conductance reactivity during an arousal task. Mediation analyses found that MCP-produced improvements in inhibitory control at post-intervention mediated program effects on reactive aggression at the one-year follow-up. Within-person analyses with the full sample (MCP and CP) found that improvements in respiratory sinus arrhythmia reactivity were associated with improvements in reactive aggression at the one-year follow-up. Together, these findings indicate that MCP is an important new preventive tool to improve embodied awareness, self-regulation, stress physiology, and observable long-term behavioral outcomes in at-risk youth. Further, children’s inhibitory control and autonomic nervous system functioning emerged as key targets for preventive intervention.

## 1. Introduction

Coping Power (CP) is a cognitive behavioral preventive intervention for children at risk for substance use and delinquency [1]. CP reduces aggressive and externalizing behavior of children prior to the onset of adolescence by effectively targeting the mediating processes of the child (hostile attributional biases, emotional dysregulation, social perspective-taking, problem-solving) and of the family (parenting) [2,3]. CP is conceptually framed by the contextual social–cognitive model of aggressive behavior [1,2], and multiple RCTs demonstrated beneficial CP prevention and treatment effects on children’s externalizing behavior problems, even at a follow-up years later [4,5,6]. Preventive effects of CP at follow-up include increased child prosocial and academic outcomes [7] and reduced rates of adolescent substance use and antisocial behavior [1,8,9].

### 1.1. Proactive and Reactive Aggression

Childhood aggression, as a predictor of later problem behaviors, such as substance use and delinquency, has been categorized into two independent dimensions: proactive and reactive [10]. While these two types of aggression often co-occur, they differ in etiology, with independent genetic influences [11] and social–cognitive patterns [12,13]. Proactive aggression has been associated with violence and antisocial personality in young adulthood, whereas reactive aggression has been associated with greater emotional lability and impulsivity, with an increased risk of substance use in adolescence [14,15,16]. Proactive aggression is instrumental and uses aggressive behaviors to obtain deliberate outcomes. Proactive aggression has been characterized as “cold-blooded,” that is, predatory, organized, and unprovoked, with a greater instrumentality orientation, while reactive aggression is explosive and “hot-blooded.” Brain imaging studies revealed that children with heightened levels of proactive aggression are marked by differing neural correlates and prefrontal and ventromedial prefrontal cortex activation [17,18,19] compared with children with reactive aggression.

CP has been effective in reducing children’s proactive aggression at post-intervention in two school-based samples of children screened for moderate-to-high rates of aggressive behavior. There was a pre–post reduction in parent-rated proactive aggression in the small effect size range (0.28) in a study of 245 elementary school boys and girls in the United States [20]. In a second study, there were small parent-rated (0.29) and moderate teacher-rated (0.57) reductions in proactive aggression for elementary school boys screened for aggression in Pakistan [21]. Moderate-sized (0.47) reductions in teacher-rated proactive aggression over a long-term three-year follow-up were found in a study that compared CP with typical school services with 241 elementary school boys and girls. Intervention effects in this follow-up study and another 6-year follow-up study [8], were also found on stable callous–unemotional traits, which were associated with proactive aggression in longitudinal research [22].

While CP was effective in consistently reducing proactive aggression in this set of randomized trials, CP’s effects on reactive aggression were more mixed in these same studies. CP had positive moderate-level effects on reactive aggression in a three-year follow-up study [4], likely due to children perceiving fewer possible provocations from others as their externalizing behavior decreased. However, there were conflicting results from the immediate post-intervention, which is when the intervention-related mechanisms should have been activated. CP produced strong reductions in reactive aggression with the boys in the Pakistan study [21], but there were no significant effects on reactive aggression in the earlier US study [20]. In this latter study, it is notable that a targeted mechanism associated with proactive aggression (outcome expectation) was influenced by the intervention but targeted mechanisms typically associated with reactive aggression, such as hostile attributions and affective dysregulation [12], were not. Thus, it appears that the targeted mechanisms associated with reactive aggression may be more easily affected by differences in the training and implementation of CP and by cultural context, and are vulnerable to not being addressed as deeply or as well as the mechanisms associated with proactive aggression. As a result, the active mechanisms of reactive aggression may need to be further targeted in more intensive ways to enhance the CP intervention effects.

### 1.2. Active Mechanisms of Reactive Aggression

**Behavior dysregulation**. Children high on reactive aggression have been associated with volatility, inattentiveness, and emotional/fear responsiveness, followed by a sense of relief [10]. Reactive aggression is also independently related to a variety of other indices, including internalizing problems, poor prosocial outcomes, and attentional problems [16,23]. Children with increased levels of reactive aggression are also marked by hyperresponsivity in the midbrain and limbic systems rather than the cortex, as children with heightened proactive aggression demonstrate [24].

**Emotional dysregulation**. Children with reactive aggression evidence high levels of anger [25,26], emotional arousal [27], and negative emotionality [25]. From a physiological perspective, reactive aggression is associated with autonomic over-arousal and increased amygdala response to social threats [28]. During an experimental anger induction task, reactive aggression (but not proactive aggression) was associated with children’s skin conductance reactivity and angry nonverbal behaviors [29]. Reactive aggression was associated with decreased SCL reactivity at the beginning of the social challenge task, then with increased SCL reactivity as the task continued. In another study, children with reactive aggression demonstrated higher skin conductance and mild cortisol decline compared with children with proactive aggression in stress-inducing tasks [30].

**Cognitive and attention dysregulation**. Children with reactive aggression often interpret social cues as hostile provocations or threats, which consequently results in heightened anger responses [31], and anger rumination has been linked with higher levels of reactive aggression in young adults [32]. In adults, anger rumination can lead to increased aggressive behavior [33], and children with high levels of reactive aggression are also associated with a reduction in executive function [34].

### 1.3. Polyvagal Theory and Reactive Aggression

Since reactive aggression is associated with greater dysregulation and differs etiologically from proactive aggression, recent studies suggest that polyvagal theory may provide a hypothesis for why reactive aggression shows resistance to intervention: reactive aggression relates to the autonomic nervous system differently than proactive aggression [35]. For children with elevated levels of reactive aggression, their dysregulated responses may be linked to poor regulation of arousal or hyperarousal of the autonomic nervous system [36].

According to polyvagal theory [37], the autonomic nervous system (ANS) automatically engages when an individual experiences threats and uncertainty, and self-regulation is linked to neurophysiological responses of the two ANS branches of the vagus nerve, namely, the sympathetic (SNS) and parasympathetic nervous systems (PNS). Polyvagal theory hypothesizes that increasing arousal is linked to the SNS, and decreasing arousal is linked to the PNS [36,38]. The SNS is responsible for increasing the metabolic responses associated with preparing for “flight or fight”, such as increased blood pressure, higher heart rate, and cardiac output, while the PNS physiologically restores and conserves energy by regulating the blood pressure, heart rate, and stress [39]. Together, these two branches (SNS and PNS) regulate arousal responses. The ANS has been considered a predictor of aggressive behavior in youth [40,41]. Impaired autonomic system reactivity (as measured by sympathetic and parasympathetic reactivity) has been correlated with increasing levels of aggressive and disruptive behavior [42,43,44].

**Skin conductance level (SCL) as a measure of increasing arousal**. The SNS prepares the body for “fight or flight” by increasing physiological arousal through increased electrodermal activity, or the skin conductance level (SCL). Thus, the SCL is a physiological signal for the stress response. Increased SCL activation has also been conceptualized as a marker for behavioral inhibition in aversive contexts (activated as fearfulness in aversive contexts) [45], whereas reduced levels of SCL reactivity are associated with higher levels of externalizing behaviors in youth [44,46]. While a low SCL has been consistently associated with aggression, this relationship is not always straightforward. Some studies found that SCL reactivity varies in children with reactive and proactive aggression [29,38]. In one study, reactive aggression was associated with decreased SCL reactivity at the beginning of a social challenge task, then with increased SCL reactivity as the task continued. Meanwhile, SCL reactivity was not related to proactive aggression [29].

**Respiratory sinus arrhythmia (RSA) as a measure of decreasing arousal**. In contrast, the activation of the PNS conserves and restores energy by reducing physiological arousal. The PNS is indexed by respiratory sinus arrhythmia (RSA), or heart rate variation, which is considered a measure of vagal tone according to polyvagal theory [37,47]. RSA functions as an index for the PNS, specifically for cardiac activity [48]. Lower RSA at baseline and higher RSA reactivity following arousal (i.e., vagal withdrawal) has been associated with greater externalizing behaviors and greater increases in delinquency over time, as well as increased prefrontal cortex dysfunction [49,50]. From the framework of polyvagal theory, higher RSA reactivity indicates the vagal brake is not engaged, and higher reactivity has been associated with anxiety, social difficulty, and problem behavior [51], while higher RSA and lower RSA reactivity are associated with prosocial behavior and emotion regulation [52,53].

### 1.4. Mindfulness Training Effects

Recent studies indicate that mind–body therapies have a wide range of positive impacts, including on the physiological response to stress [54]. Mindfulness training is one form of mind–body practice [55,56]. Mindfulness is described as the practice of bringing non-judgmental awareness to the present moment [56]. Mindfulness training practices focus on noticing one’s breathing, bodily sensations, thoughts, and moment-to-moment experiences through an accepting, non-judgmental lens. Paying attention in this way can help to increase one’s focus on the present moment (rather than ruminating on the past or future), reduce negative thought and emotion patterns, and create space between emotional reactions and behavioral responses [55,56].

Mindfulness training in the school setting was shown to improve child behavior outcomes, such as attentional control, emotion regulation, and impulse control [57,58,59]. These studies indicate that integrating mindfulness training into Coping Power can improve the active mechanisms of reactive aggression and strengthen program effects on children’s behavior and stress physiology.

**Mindfulness effects on behavior dysregulation**. Mindfulness training improves behavior outcomes for the social development of elementary students [60] and adolescent impulsivity [61]. Mindfulness training also showed improved behavioral outcomes for children with externalizing behavior disorders [62].

**Mindfulness effects on emotion dysregulation**. Mindfulness training improves emotional outcomes by affecting externalizing anger in adolescents [63] and self-regulation in young adults [64]. Brain imaging studies indicate that mindfulness training is linked to improvements in the density of brain regions linked to emotion regulation [65].

**Mindfulness effects on cognitive and attention dysregulation**. Mindfulness training improves cognitive and attention outcomes and was shown to decrease rumination [59,62]; increase cognitive outcomes, such as cognitive flexibility [60] and attention [66]; and improve attention outcomes in children with attention disorders [67].

### 1.5. Targeting Active Mechanisms of Dysregulation with Mindful Coping Power

Given the demonstrated effects of mindfulness training on the active mechanisms of reactive aggression, it was hypothesized that incorporating mindfulness practices into Coping Power would strengthen the program’s effects on reactive aggression and improve children’s ANS functioning. The Mindful Coping Power (MCP) program, which integrates mindfulness into the cognitive behavioral Coping Power program, was developed to test this hypothesis [3].

A recent randomized comparative effectiveness study of MCP versus CP found that for children with high levels of reactive aggression, MCP yielded significantly stronger effects than CP on children’s self-reported dysregulation [68]. By targeting the active mechanisms of attentional, cognitive, behavioral, and emotional dysregulation present in high levels of reactive aggression, MCP optimized the benefits of the cognitive–behavioral intervention with anticipated effects on the underlying mechanisms of reactive aggression [3]. Of note, the strongest effects were on children’s self-reported internal experiences, with fewer effects on children’s overt behavior, as observed by teachers. Based on these comparative findings, the authors hypothesized that children’s improved sense of internal self-regulation and embodied awareness in MCP versus CP at post-intervention may lead to greater improvements in the observable behavior over time for children in the MCP group. This was hypothesized because children’s increased internal awareness and self-regulation at post-intervention, if maintained or strengthened with ongoing mindfulness practice, may lead to more prosocial and fewer hostile–aggressive observable behaviors over time. It was also hypothesized that MCP improved children’s ANS functioning at post-intervention, corresponding with their perceived improvements in internal self-regulation. If found, improvements in children’s ANS functioning at post-intervention are also expected to contribute to improved observable child behavior over time.

### 1.6. The Current Study

The current study examined the above hypotheses. Specifically, the following research questions guided this study:

(1) Does Mindful Coping Power (MCP) produce better child behavioral outcomes at a 1-year follow-up than Coping Power (CP), as measured by teacher-rated child reactive aggression, externalizing behavior problems, proactive aggression, and social skills?
(2) Does the Coping Power preventive intervention (full sample with both MCP and CP) have a beneficial impact on children’s physiological stress reactivity (SCL and RSA reactivities) from pre- to post-intervention? If so, does MCP produce stronger beneficial effects on children’s stress physiology than CP?
(3) Do intervention-produced improvements in children’s stress physiology (SCL and RSA reactivities) and perceived self-regulation and mindful awareness (total dysregulation, inhibitory control, breath awareness) from pre- to post-intervention mediate improvements in child behavioral outcomes at a 1-year follow-up (as measured by child reactive aggression, externalizing behavior problems, proactive aggression, and social skills)?

## 2. Materials and Methods

### 2.1. Participants

**Participating schools**. This study is a follow-up from a previously published study [68], which reported the results of a preventive trial that compared standard CP versus MCP child behavioral outcomes at pre- and post-intervention. Five elementary schools in Tuscaloosa, Alabama, were recruited for this study (four participated in the first annual cohort and a fifth was added in the second annual cohort). The schools varied in terms of sociodemographic characteristics, including families enrolled in free or reduced lunch (ranging from 32% to 76%) and each child’s race (with Black or African American the most prevalent, ranging from 92% to 32%). To control for sociodemographic characteristics, participants were randomly assigned to MCP or CP within each school.

**Child participants**. The full recruited sample included 102 children with elevated levels of reactive aggression, as well as their parents and teachers. Child participants were identified through teacher screener ratings completed at the end of the fourth grade so that recruited children could participate in the intervention during their fifth-grade year. The CP preventive intervention was designed to target preadolescent children immediately prior to the middle school transition when substance use initiation risk increases [69].

To identify children with elevated reactive aggression, fourth-grade teachers completed the Teacher Report of Reactive and Proactive Aggression [70] on all classroom students. Ratings were compiled across the participating schools and divided into quartiles to identify an empirical cut-off score that reflected the top quartile of reactive aggression in fourth-grade students. An empirical cut-off score of 8 was used, which was consistent with previous CP studies in which children with teacher-rated reactive aggression scores at or above this level had elevated parent-rated externalizing problems (at-risk or clinical range) on the Behavior Assessment Scale for Children [71].

All enrolled child participants were offered an opportunity to provide stress physiology data and to have their sixth-grade teacher provide one-year follow-up data (which was not part of the initial study consent), with parent consent. The current analyses included the subset of participants who provided either or both forms of additional data. Table 1 summarizes the characteristics of the children with complete one-year follow-up data (*n* = 80), SCL data (*n* = 46), and RSA data (*n* = 45) compared with the full sample.

The current sample was predominantly Black/African American (88%, *n* = 70), male (64%, *n* = 51), and the majority of families were from a lower-income household (66% had a household income of less than USD 30,000). No significant differences were observed between participants in the intent-to-treat sample (*n* = 102) and those in the current analyses on key baseline or demographic variables. Compared with the intent-to-treat sample, there were trend effects (*p* < 0.10) for children from higher-income families to be less likely to have one-year follow-up data, and for children in CP to be less likely to have SCL data.

### 2.2. Procedure

All study procedures were approved by the University of Alabama Institutional Review Board. Data on the participants was collected in multiple waves, with participant data gathered at three time points. Additional details about full study procedures can be found in a prior publication [68].

**Participant recruitment and retention**. Rising fifth-grade students were recruited from five schools across two annual cohorts. A total of 638 fourth-grade students were screened for study participation. Of these, 428 scored below the empirical cut-off for teacher-rated reactive aggression. One child with an eligible screener score was excluded due to a language barrier that could not be addressed with local resources. The remaining families were contacted in random order until the total number of intervention slots at each school had been filled. Six families declined study participation (the most common reasons were that the child already received services elsewhere or a lack of perceived need). The children who declined did not significantly differ regarding baseline characteristics from those enrolled.

One hundred and eight children were initially enrolled in the study. Five of these children moved to different schools prior to starting fifth grade. The remaining 103 participants were randomly assigned to either the MCP or CP group at their school in yoked pairs, stratified by reactive aggression screener score and demographic characteristics (to create equivalent MCP and CP groups at each school). One child withdrew from the study after participating in one session (due to a perceived lack of need and social concerns), which resulted in a total sample of 102 child participants, as well as their parents and teachers. This resulted in a sample of 52 children randomly assigned to MCP and 50 to CP.

Four schools participated in both cohorts and a fifth school was added for cohort two. This led to a total of 44 participants in cohort 1 and 58 participants in cohort 2.

Pre-intervention data were collected from parents and children at the start of fifth grade and from teachers four to six weeks into the school year (to allow teachers to gain familiarity with the children’s behavior). The intervention began following the completion of the time 1 assessments. Post-intervention data were collected at the end of fifth grade for teacher data and the month following fifth grade for parents and children.

Several months after completing the original pre–post study, families were contacted and offered an opportunity to participate in an additional one-year follow-up assessment. For eighty participants, one-year follow-up data were collected at the end of sixth grade. Reasons for non-participation given by the remaining 23 participants included the following: 8 did not respond to multiple attempts to reach them, 5 declined, 5 indicated a willingness to participate but did not follow through, 3 offered to complete a mailed packet but did not return it, and 2 moved out of the area.

The administration of parent, child, and teacher assessments was blinded to the condition. For each child assessed, teachers received USD 10, and for each assessment time point, parents received USD 50 and the children received USD 10.

**Intervention**. This study compared two active preventive interventions, namely, MCP and CP. Both interventions included the same number of sessions (25 child group and 10 parent sessions) and utilized curricula with specified objectives and activities at each session. The child group sessions lasted 45 min, met weekly, and were held during the school day in a private meeting space. The parent group sessions lasted 60 to 90 min, met biweekly or monthly, and were held in the mornings and evenings in a central location near the school.

**Coping Power**. CP is an evidence-based cognitive behavioral preventive intervention for youth at risk for substance use, delinquency, and other disruptive behavior disorders [72,73]. Drawing from a cognitive–behavioral framework, CP teaches emotion coping and social–cognitive skills to children and positive parenting and self-care skills to parents. The child program topics include setting personal goals, emotional awareness, coping with anger, problem-solving, perspective-taking, prosocial peer affiliations, and resisting peer pressure [72]. The parent program topics include supporting children’s academic learning, strengthening parent–child and family relationships, managing parenting stress, setting household expectations and rules, praising, ignoring, developing effective discipline techniques, problem-solving as a family, and future planning [73].

**Mindful Coping Power**. MCP is a novel adaption of CP that integrates mindfulness into an evidence-based intervention [3]. MCP was shown to enhance the effects on children’s anger modulation and perceived self-regulation [68]. All of the core content of CP is included in MCP. Three distinct modifications were made to distinguish MCP from CP. First, MCP included additional *mindfulness-only sessions*, in which both the child and parent MCP programs introduced the theory and practice of mindfulness. For example, mindfulness was defined as “training your brain” to pay attention to the present moment more fully, which was called “noticing right now.” Second, MCP incorporated *mindfulness in every session*. Each session started and ended with 1–2 mindfulness practices (e.g., the ringing of a chime, breath awareness, yoga poses, body scan, mindful eating, mindful listening, thought awareness, or “Feel and Spread the Good Vibes” compassion practices). Children and parents also completed these mindfulness practices at home between sessions. Third, MCP *integrated mindfulness into current CP activities*, which included identifying early physiological cues of anger with body awareness practice, perspective-taking situations with compassion practice, and coping with anger with thought awareness practices for both the parent and child. For greater detail about how MCP incorporates mindfulness into CP, see Miller and colleagues [3].

CP did not have any intervention content that was not also in MCP. Each condition had the same number of sessions. An effort was made to keep the length of sessions consistent across the two conditions. Due to the added mindfulness practices in MCP and mindfulness-only sessions, more time was spent on some standard program elements in CP than in MCP (e.g., reviewing progress toward weekly personal goals and setting new goals, personal sharing related to intervention topics, and opportunities to practice new skills in session). Some topics were extended across more than one session in CP to account for the mindfulness-only sessions in MCP without having any content in CP that was not in MCP.

**Interventionists**. There were five primary group leaders. Each implemented both versions of the intervention at their assigned school (MCP and CP). These leaders were doctoral (*n* = 2) or master’s (*n* = 3) level clinicians with considerable experience implementing Coping Power, as well as training in Mindfulness-Based Stress Reduction (MBSR) and maintenance of regular mindfulness practice. MBSR is a standardized mindfulness training program that incorporates mindfulness meditation, body scanning, and simple yoga postures to obtain depth practice in moment-to-moment, non-judgmental awareness [74]. Four of the leaders were female and one was male. Four of the leaders were Caucasian and one identified as more than one race. Master’s and advanced undergraduate students served as co-leaders to provide additional group oversight and behavior support. They received CP and mindfulness training prior to co-leading the intervention groups.

### 2.3. Measures and Tasks

**Behavioral Measures**.

The behavioral measures in this study were the same as the previously published study, which provides greater detail on each measure [68].

Measures used in the current study included the Teacher Report of Reactive and Proactive Aggression (TRRPA; [70]), which was used as a screener to identify students with elevated reactive aggression and as an outcome measure; the Behavior Assessment System for Children (BASC; [71,75]), which is a broadband teacher-reported measure with composite scores for externalizing behavior problems and social skills; the Abbreviated Dysregulation Inventory (ADI; [76]), which measures children’s perceived affective, behavioral, and cognitive dysregulation; the Early Adolescent Temperament Questionnaire (EATQ; [77]), which includes a child self-reported measure of inhibitory control; and the Scale of Body Connection (SBC; [78]), which measures breath and body awareness. Additionally, as described below, the current analyses utilized physiological data gathered to measure the skin conductance level (SCL) and cardiac respiratory sinus arrhythmia (RSA) using data recorders during a decision-making card task, namely, the Iowa Gambling Task.

**Physiological Measures**.

**RSA data**. RSA data were obtained using a BioLog™ physiological data recorder, which measures the participant’s heart rate and interbeat intervals with the placement of an electrode each at the collarbone and behind the left knee, with a reference electrode placed on the right side of the neck. The RSA was derived using the Porges–Bohrer method using the CardioBatch and CardioEdit computer programs [79,80].

First, interbeat interval data were cleaned by hand-editing participants’ heart rate data in the CardioEdit program. Second, outliers or artifacts were reviewed as data points (i.e., errors in the heart rate data that were likely due to the digitizing process or physiological anomalies). Outliers were identified and edited (using standard methods, such as averaging, combining, and dividing). Files in which 5% or more of the data points required editing were removed from the sample. After cleaning, the CardioBatch program was used to extract the RSA from one of the predominant rhythms exhibited in the participants’ heart period series. The mean RSA was calculated for each of the five blocks.

**SCL data**. SCL data were obtained by placing electrodes on the volar surface of the distal phalanges (first and third fingers) of the non-dominant hand. The SCL data were processed and artifact removal was completed through the use of Ledalab [81], which is a Matlab-based software for the analysis of the SCL data. Each SCL file was scanned for outlier data points relative to adjacent data points. These outlier points were replaced by data that were more consistent with the surrounding data.

**Iowa Gambling Task (IGT)**. The IGT is a psychological task used to measure decision-making and to elicit an affective response by varying the rewards and penalties for selecting certain card decks. The IGT has been used to examine other physiological and disorder data, including brain lesions, alcohol disorders, violence and aggression, and gambling and impulse control disorders [82,83,84,85,86]. In the task, participants are not instructed about the uncertain nature of the task and are unlikely to recognize the probability of receiving positive and negative reinforcement across the different card decks; thus, participants make decisions under ambiguous conditions, with the participants’ responsiveness to emotional cues from previous card draws contributing to later advantageous task decisions [87,88,89].

Prior to the IGT, children first viewed a neutral stimulus (a two-minute scenery video) to permit the necessary time to acclimatize to the physiological equipment with minimal emotional response, language, or movement. Values for the baseline RSA and SCL were obtained during this neutral stimulus period. Following this neutral stimulus period, participants received instruction for and played the Iowa Gambling Task on a computer screen (IGT; [82]). In the task, participants are presented with four decks (varying in penalty and reward levels) and told to win as much game money as possible. Participants complete five, 5 min blocks with 20 cards, for a total of 100 cards. The SCL and RSA reactivities were assessed during the first block for this study, given that the first blocks have more uncertainty and ambiguity [90,91]. The SCL and RSA reactivities were computed as the change from baseline (neutral stimulus) to block 1 of the IGT.

### 2.4. Statistical Approach

Statistical Analysis Software (SAS/STAT 15.1) [92] was utilized to conduct the analyses for this study.

**Research questions 1 and 2** were examined using mixed-effects models. For research question 1, the outcomes of interest were the teacher-rated child social skills, externalizing behavior, reactive aggression, and proactive aggression, measured at baseline and the 1-year follow-up. The SCL and RSA reactivities, measured at baseline and post-intervention, were the dependent variables of interest for research question 2. The fixed effects included the *time*, *treatment assignment* (CP = 0, MCP = 1), and *interaction* between these two. The presence of the interaction allowed for the estimation of the time slopes for each condition separately, as well as testing whether the time slope in the MCP condition significantly differed from that in the CP condition on average. Both CP and MCP were delivered in group settings. Likelihood ratio tests examined whether it was necessary to account for nesting at the intervention-group level. The tests were insignificant. Therefore, the random effect included a random intercept only at the individual level. Models were estimated with a restricted maximum likelihood. A Kenward–Roger adjustment was used to mitigate the small-sample bias. The effect size was estimated using Equation (8) in Morris [93]. All analyses included dummy variables to control for school differences. The models for SCL and RSA also controlled for humidity and temperature.

**For research question 3**, mediation analyses were conducted using the ANCOVA approach, which involved two regression equations [94]. In the first equation, the mediator measured at post-intervention was regressed on the treatment assignment, adjusting for the outcome and the mediator measured at baseline. In the second equation, the outcome measured at the 1-year follow-up was regressed on the mediator measured at post-intervention, adjusting for the treatment assignment, and the outcome and the mediator measured at baseline. The significance of the indirect effect was determined using a confidence interval generated with PRODCLIN [95]. This was repeated for each potential mediator and outcome variable when the following conditions were satisfied: the treatment condition had a significant effect on the potential mediator at post-intervention, and the potential mediator had a significant effect on the outcome variable at the one-year follow-up, controlling for the treatment condition. The SCL and RSA reactivities were explored as potential mediators, as well as the child’s self-reported measures of self-regulation and mindful awareness, which showed greater improvement with MCP in prior pre–post analyses [68]. Outcome variables included externalizing behavior problems, reactive aggression, and social skills.

Mixed-effects models were also used to examine the within-person association between the SCL and RSA reactivities and each behavioral outcome variable (reactive aggression, externalizing behavior problems, proactive aggression, and social skills). For example, it was hypothesized that an increase in the RSA reactivity between baseline and post-intervention would be associated with an increase in externalizing behavior problems between baseline and the 1-year follow-up. Other time-varying variables included reactive aggression, proactive aggression, social skills, and a variable *time* to capture a secular trend. All time-varying independent variables were person-mean-centered to estimate their within-subject association with the behavioral outcome variable (e.g., externalizing behavior). In addition to race and gender, the model incorporated individual-specific means of the time-varying independent variables as (time-invariant) individual-level variables. Individual-level variables captured between-person associations with the child’s behavioral outcome variable (e.g., externalizing behavior).

## 3. Results

### 3.1. Results

#### 3.1.1. Research Question 1

Does Mindful Coping Power (MCP) produce better child behavioral outcomes at a 1-year follow-up than Coping Power (CP), as measured by teacher-rated child reactive aggression, externalizing behavior, proactive aggression, and social skills?

Table 2 summarizes the mean scores for each teacher-reported child behavioral outcome at pre-intervention and the 1-year follow-up, as well as the effect size estimates comparing outcomes from MCP and CP. The effect sizes were computed using the pooled pretest standard deviation [93]. The effect sizes observed from pre-intervention to the 1-year follow-up each favored MCP relative to CP and were as follows: 0.57 for social skills, −0.46 for reactive aggression, −0.36 for externalizing behavior problems, and −0.26 for proactive aggression.

**Figure 1:**
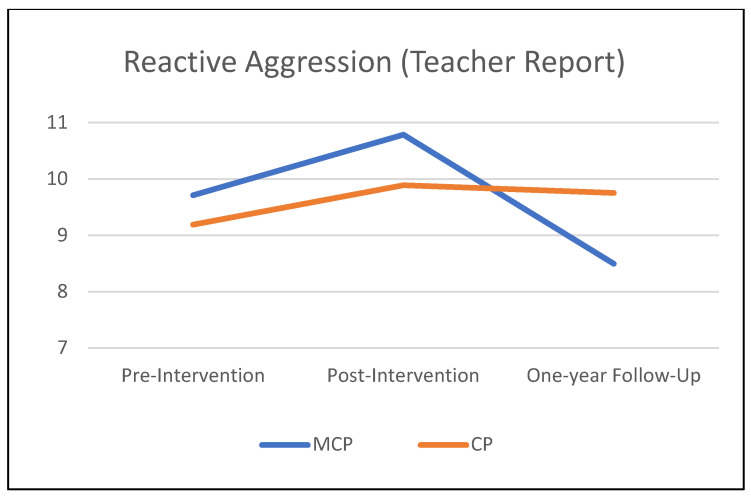
One-year follow-up effects on children’s reactive aggression.

**Figure 2:**
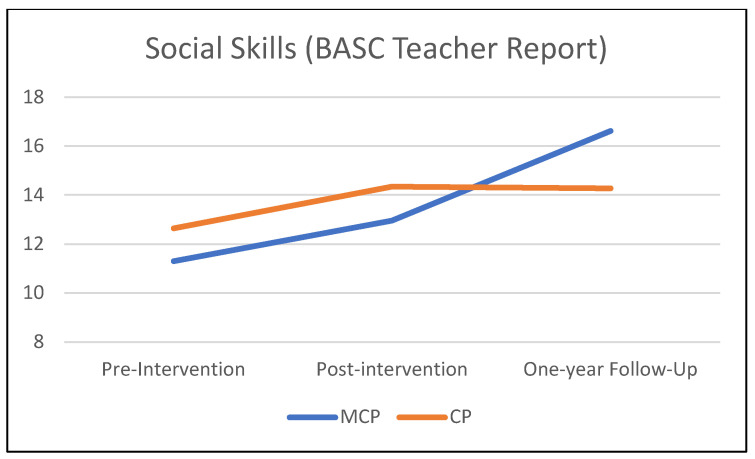
One-year follow-up effects on children’s social skills.

**Figure 3:**
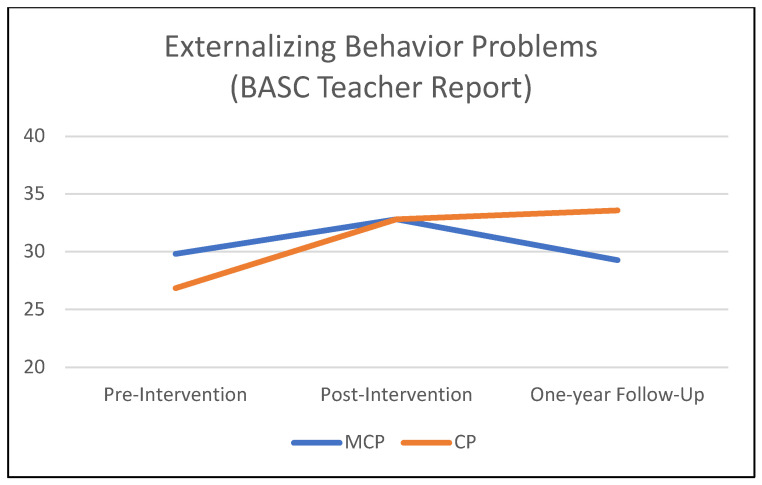
One-year follow-up effects on children’s externalizing behavior problems.

Table 3 summarizes the results of the mixed-effects models comparing the MCP and CP interventions on teacher-rated child behavioral outcomes from pre-intervention to the one-year follow-up. Figure 1, Figure 2 and Figure 3 illustrate these findings. A significant *time* × *condition* effect indicated that MCP produced significantly stronger improvement than CP in children’s social skills (*p* = 0.037). The *time* × *condition* effect was marginally significant for reactive aggression (*p* = 0.057), with more improvement in the MCP group than in the CP group. Children in the MCP group showed a significant increase in social skills (*p* < 0.01) on average, and a marginally significant decrease in reactive aggression (*p* = 0.055). Meanwhile, the average change in the CP group was non-significant for both social skills and reactive aggression (*p* > 0.20). Children in the CP group showed a significant worsening of externalizing problems (*p* =.041) on average, while the change in the MCP group was non-significant (*p* = 0.884). However, the *time* × *condition* interaction did not attain statistical significance at 5% for externalizing problems (*p* = 0.109). In terms of proactive aggression, children in the MCP group showed a significant decline (*p* =.002) on average, while the change in the CP group was non-significant (*p* = 0.120). The *time* × *condition* interaction, however, did not attain statistical significance for proactive aggression (*p* = 0.294).

#### 3.1.2. Research Question 2

Does Coping Power (full sample with both MCP and CP) have a beneficial impact on children’s physiological stress reactivity (SCL and RSA reactivities) from pre- to post-intervention? If so, does MCP produce stronger beneficial effects on children’s stress physiology than CP?

**SCL and RSA Reactivity**. For the stress reactivity measures, the SCL and RSA reactivities were computed as the change from baseline (neutral stimulus) to block 1 of the IGT. The difference between the two time points, which reflects reactivity, was used to determine a change in children’s stress physiology, either increasing or decreasing depending on the measure (e.g., skin conductance or cardiac respiratory sinus arrhythmia). Since the physiological assessment was optional and the authors employed stringent cut-off scores for the usable data (i.e., data with 5% or greater data editing was removed), the number of cases was reduced, with SCL and RSA reactivity scores for 46 and 45 participants, respectively.

Table 4 summarizes the mean scores for the SCL and RSA reactivities at pre- and post-intervention, as well as effect size estimates comparing outcomes in the MCP and CP conditions. The effect sizes were 0.66 for the SCL reactivity and −0.25 for the RSA reactivity, each favoring MCP.

Table 5 summarizes the results of the mixed-effects models comparing the MCP and CP intervention conditions on the SCL and RSA reactivities from pre- to post-intervention. There was a significant difference between MCP and CP for the SCL reactivity (*p* = 0.027). On average, children in the CP condition showed a decline in SCL reactivity at post-intervention (*p* = 0.005), while the change in the MCP condition was not significant (*p* = 0.830). The effects on RSA reactivity were not significant.

#### 3.1.3. Research Question 3

Do intervention-produced improvements in children’s stress physiology (SCL and RSA reactivities) and perceived self-regulation and mindful awareness (total dysregulation, inhibitory control, breath awareness) from pre- to post-intervention mediate improvements in child behavioral outcomes at a 1-year follow-up (as measured by reactive aggression, child externalizing behavior problems, proactive aggression, and social skills)?

Inhibitory control emerged as the primary mediator of interest. SCL reactivity, total dysregulation, and breath awareness did not show significant mediation effects on reactive aggression or social skills. RSA reactivity was not assessed as a mediator, and externalizing behavior problems and proactive aggression were not included as outcome measures due to not meeting the criteria for inclusion in mediation analyses.

Table 6 presents results from the mediation analysis with inhibitory control as the mediator, reactive aggression as the outcome, and treatment assignment as the independent variable. The effect of MCP (vs. CP) on inhibitory control at post-intervention was marginally significant at 10% (*b* = 0.217, *p* = 0.070). The effect of inhibitory control at post-intervention on reactive aggression at the 1-year follow-up was significant at 5% (*b* = −1.614, *p* = 0.024). The indirect effect of MCP (vs. CP) on reactive aggression at the 1-year follow-up through inhibitory control at post-intervention was estimated by the product of 0.217 and −1.614. Children in the MCP condition had higher inhibitory control at post-intervention on average, which, in turn, was associated with lower reactive aggression at the 1-year follow-up. Using PRODCLIN, it was suggested that the indirect effect was significant at 10% (90% *CI* = [−0.827, −0.009]). The models controlled for child demographics, including age, sex, and race.

For the analysis of within-person associations, the main finding of interest was found for the RSA reactivity and externalizing behavior problems. As shown in Table 7, the mixed-effects analyses indicated a within-person association between the RSA reactivity and teacher-reported externalizing behavior problems. An increase in RSA reactivity between baseline and post-intervention was associated with an increase in externalizing behavior problems between baseline and the 1-year follow-up (*p* < 0.01). This model was estimated with an interaction between the RSA reactivity and MCP to test for differential intervention effects. It was not significant, and thus, the model was re-estimated without the interaction, as reported in Table 7 below. Although it is not of main interest, there was a significant within-person association between reactive aggression and externalizing behavior problems (*p* < 0.01). An increase in reactive aggression from baseline was associated with an increase in externalizing behavior problems from baseline (*p* < 0.01). On the other hand, proactive aggression and social skills were not within-person-associated with externalizing behavior problems (*p* = 0.549 and 0.533, respectively).

## 4. Discussion

### 4.1. MCP Effects on Children’s Long-Term Behavioral Outcomes

MCP was developed to enhance CP effects on reactive aggression by incorporating mindfulness training into the intervention to target the active mechanisms of reactive aggression more directly. At post-intervention, Boxmeyer and colleagues [68] found that MCP improved children’s self-reported embodied awareness, self-regulation, and anger modulation relative to CP, but had fewer comparative effects on parent- and teacher-reported behavioral outcomes, including reactive aggression. The current study examined teacher-reported child behavioral outcomes one year after the intervention ended to appraise the hypothesis that MCP-produced improvements in children’s internal experiences at post-intervention would yield improvements in children’s observable behavior later in development. Indeed, the results of the current study partially support this hypothesis. One year after the intervention, children in the MCP group showed improvements in teacher-reported prosocial behavior and a statistical trend for the reduction in reactive aggression relative to children in the CP group.

As a preventive intervention, one of the main goals of CP is to alter the developmental trajectory of at-risk youth as they transition to adolescence. Preadolescents who exhibit high levels of aggression are at risk for a range of negative outcomes across the developmental cascade, including violence, delinquency, substance abuse, and academic difficulties [16,96]. Positive program impact has not been defined solely as an overall net reduction in aggression and externalizing behavior, especially when fall-to-spring teacher ratings are used and teachers have greater exposure to children’s behavioral problems throughout the school year. Positive program impact has also been defined as an attenuation in the slope of the progression toward negative adolescent outcomes [68,97]. Thus, it is even more noteworthy that children in the MCP group exhibited higher levels of social skills, lower proactive aggression, and a trend for lower levels of reactive aggression at the end of their first year of middle school, one year after completing the intervention, compared with pre-intervention levels early in fifth grade. Moreover, MCP appears to have added a benefit beyond the already established, evidence-based CP in terms of improving children’s long-term social skills and the tendency to reduce reactive aggression. The hypothesis was upheld that the MCP-produced improvements in children’s internal awareness and perceived self-regulation at post-intervention would eventually lead to comparative improvements in their observable behavior, even though the MCP versus CP impacts on observable behavior were not consistent immediately post-intervention [68]. This additional one-year follow-up data underscores the idea that MCP is an important enhancement of CP that can maximize the program’s downstream effects.

Additional analyses examined whether these long-term improvements were attributable to MCP-produced changes in children’s stress physiology and children’s self-reported embodied awareness, anger modulation, and self-regulation.

### 4.2. MCP Effects on Children’s Autonomic Nervous System Functioning

MCP produced stronger effects than CP on children’s autonomic nervous system functioning from pre- to post-intervention. MCP had a significant effect on the children’s SCL reactivity (ES = 0.66) and a smaller, non-significant effect on the RSA reactivity (ES = −0.25).

Children’s poor emotional regulation can result from overactivity of the sympathetic nervous system (SNS; prepares for “fight or flight”) and a diminished ability of the parasympathetic nervous system (PNS) to dampen that arousal and bring children back to their initial level of arousal. Extreme difficulties regulating arousal can cause aggressive children to experience intense anger and “hot” angry cognitions, which then interfere with their adaptive information processing and leads to reactive aggression [31,98,99].

Higher SCL activation has been conceptualized as facilitating behavioral inhibition through the production of fear and anxiety. Lower levels of SCL reactivity have been associated with higher levels of youth problem behaviors [44,46,100]. Some studies found that SCL reactivity relates differently to reactive and proactive aggression [29,38] and at different phases of an anger-inducing game [29]. The present study examined the SCL reactivity in the transition from baseline to the first block of an arousal task and found that, on average, children in the CP condition showed a decline in SCL reactivity from pre- to post-intervention, while the change due to the MCP condition was not significant. This indicates that children in the MCP group maintained better regulation of arousal during an emotionally arousing decision-making task from pre- to post-intervention than those in CP. Improving the regulation of physiological arousal, or at least attenuating declines in arousal regulation, has the potential to have a lasting impact on functioning across the developmental cascade.

RSA is a measure of the parasympathetic nervous system, which conserves and restores energy. Higher RSA at rest indicates the vagal brake is engaged and has been associated with prosocial behavior, while higher RSA reactivity indicates the vagal brake is not engaged and has been associated with anxiety, social difficulty, and behavior problems [37,41,49,101,102,103]. In the current study, there was a small but non-significant effect favoring MCP on the RSA reactivity. Even small improvements in the children’s RSA reactivity may have lasting benefits on their emotional regulation and behavior, which was supported by the within-person analyses in the full sample discussed below.

### 4.3. Mediational Analyses

Inhibitory control emerged as a primary mediator of interest. MCP-produced improvements in inhibitory control at post-intervention mediated the program’s trend for effects on reactive aggression at the one-year follow-up. Inhibitory control is an important aspect of executive functioning, which entails the effortful or willful control of behavior, including both approach and avoidance behavior [104]. Problems with inhibitory control are common in youth with aggressive behavior [105,106], predict later externalizing problems and substance use [107,108,109], and may moderate the relationship between negative emotionality and externalizing behavior problems [110]. Boxmeyer and colleagues [68] found that children in the MCP group showed greater improvements in inhibitory control than children in CP, making MCP an important tool for preventing and reducing a range of negative adolescent outcomes in at-risk youth.

Within-person analyses found that improvements in RSA reactivity were associated with improvements in reactive aggression at the one-year follow-up in the full sample (both treatment conditions combined). As described above, RSA is increasingly seen as an important biomarker of emotion dysregulation and various forms of externalizing and internalizing psychopathology [49]. These findings indicate that both inhibitory control and RSA are important targets for preventive intervention to improve children’s physiological, behavioral, and emotional regulation. MCP is a valuable new prevention tool, particularly to improve inhibitory control in children exhibiting reactive aggression.

Other potential mediators did not meet analytic requirements or show significant effects, including the SCL reactivity, perceived self-regulation measured using the ADI, or body awareness measured using the SBC. In the original pre–post sample [68], the Child and Adolescent Mindfulness Measure [111] was also collected but was not as sensitive to differences between MCP and CP as was the SBC measure of body and breath awareness. For this reason, the SBC was included as a potential mediator in the current analyses rather than the CAMM. The differences in the performance of the CAMM and SBC with this sample may have been because both CP and MCP have significant components on thought awareness, while MCP invests much more heavily than CP on present moment breath and body awareness.

### 4.4. Study Limitations

This study employed a rigorous design in that it directly compared MCP with standard CP (which already has an extensive evidence base) in a randomized controlled trial. Teachers were blind to the condition and the same clinicians implemented both the MCP and CP groups at each school, thus controlling for clinician characteristics. One limitation of the study, however, was that it did not include a no-treatment control group. As described above, previous studies found that without a preventive intervention, such as Coping Power, youth with elevated anger and aggression are at risk for an increasing trajectory of negative outcomes into adolescence and adulthood [16,96]. Attenuating this trajectory toward negative outcomes is seen as a success (i.e., reducing the slope of increases in externalizing behavior, reactive aggression, social skill deficits, and associated behaviors such as substance use), as well as net improvements in these outcomes from baseline. If this study had included a no-treatment control group, the full preventive benefits of MCP and CP may have been characterized more comprehensively.

Another study limitation was attrition from the intent-to-treat sample. Collection of the teacher data at the one-year follow-up was not part of the initial study design, thus participants had to consent to this separately. About 78% percent of the original intent-to-treat sample provided one-year follow-up data. Overall, the follow-up sample was reflective of the intent-to-treat sample, except for a trend toward less retention of higher-income families (in an already lower-income sample, with the majority of families having an annual income of less than USD 30,000 per year). Participants also consented to the collection of physiological data separately and high standards were set for the quality of the SCL and RSA data to be included in analyses. Thus, only 44% of the initial sample was included in the analyses of intervention effects on the SCL and RSA reactivities. These children were generally representative of the full sample, except for a trend effect for children in the CP group to be less likely to have usable SCL data than those in the MCP group.

The original sample of 102 participants was powered as a pilot and feasibility study to estimate the comparative effects of MCP versus CP in preparation for a large-scale trial. Since fewer participants provided stress physiology and one-year follow-up data in the current analyses, the power to detect small effect sizes was even more limited. For example, the trend effect favoring MCP on children’s reactive aggressive behavior (ES = −0.46) and externalizing behavior at the one-year follow-up (ES = −0.36), and other outcomes with smaller effect sizes, may have been statistically significant with a larger sample. Thus, an important future direction is to explore the effects of MCP in a larger, more adequately powered sample.

A final limitation was the use of a nonsocial stressor procedure to evoke uncertainty and emotional arousal, namely, the IGT, and to examine the change in children’s physiological functioning. The IGT assesses participants’ affective decision-making under conditions of uncertainty and ambiguity about the consequences of choices on a card selection task and produces resultant physiological arousal evident in SCL and RSA reactivities [100]. Other paradigms are available that measure vagal withdrawal and skin conductance reactivity during simulated social interactions designed to elicit social appraisals and emotional arousal similar to real-life rejecting or conflictual interpersonal interactions, e.g., [112]. It would be beneficial to study the effects of MCP using such a typical social stressor paradigm in the future.

## 5. Conclusions

This paper provides further evidence that targeted preventive interventions delivered in elementary school can significantly alter the developmental trajectory of at-risk youth, impacting not only the children’s behavior but also their autonomic nervous system functioning. CP is one of the leading evidence-based preventive interventions for at-risk aggressive youth. MCP is a novel adaptation of CP that incorporates mindfulness training to maximize the program’s effects on reactive aggression. The current study built upon prior findings stating that MCP improves children’s self-reported anger modulation, self-regulation, and embodied awareness compared with CP at post-intervention. The current study found that these post-intervention improvements were accompanied by improvements in children’s autonomic nervous system functioning and that MCP produced long-term benefits to children’s social skills and trends for the reduction in reactive aggression (as rated by teachers) one year after the intervention. Mediational analyses indicated that MCP produced long-term effects on children’s reactive aggression by improving children’s inhibitory control. Based on within-subjects analyses, improving children’s RSA reactivity is also an important mechanism for improving children’s long-term externalizing behavior outcomes. Overall, these findings highlight that MCP is an important new preventive intervention for at-risk youth, in particular, those with emotional and behavioral dysregulation. Future studies are needed to examine the effects of MCP in large-scale trials.

## Figures and Tables

**Table 1:** Participant characteristics.

	**Cases with Complete** **1-Year Follow-Up Data**			**Full Intent-to-Treat** **Sample**		
	**Overall** **(*n* = 80)**	**MCP** **(*n* = 43)**	**CP** **(*n* = 37)**	**Overall** ***(n* = 102)**	**MCP** **(*n* = 52)**	**CP** **(*n* = 50)**
	*m* (*sd*)	*m* (*sd*)	*m* (*sd*)	*m* (*sd*)	*m* (*sd*)	*m* (*sd*)
**Child’s age**	10.0 (0.46)	10.0 (0.49)	10.0 (0.44)	10.0 (0.48)	10.0 (0.49)	9.9 (.47)
**Child’s reactive aggression 4th grade**	11.3 (2.44)	11.2 (2.47)	11.3 (2.45)	11.2 (2.37)	11.2 (2.39)	11.2 (2.37)
	*n* (%)	*n* (%)	*n* (%)	*n* (%)	*n* (%)	*n* (%)
**Child’s gender**						
Male	51 (63.7%)	28 (65.1%)	23 (62.2%)	62 (60.8%)	33 (63.5%)	29 (58.0%)
Female	29 (36.3%)	15 (34.9%)	14 (37.8%)	40 (39.2%)	19 (36.5%)	21 (42.0%)
**Child’s ethnicity**						
Hispanic or Latino	2 (2.5%)	1 (2.3%)	1 (2.7%)	3 (2.9%)	1 (1.9%)	2 (4.0%)
Not Hispanic or Latino	72 (90%)	39 (90.7%)	33 (89.2%)	91 (89.2%)	47 (90.4%)	44 (88.0%)
Unknown or not reported	6 (7.5%)	3 (7.0%)	3 (8.1%)	8 (7.8%)	4 (7.7%)	4 (8.0%)
**Child’s race**						
Black or African American	70 (87.5%)	38 (88.4%)	32 (86.5%)	89 (87.3%)	47 (90.4%)	42 (84%)
White or Caucasian	4 (5.0%)	2 (4.7%)	2 (5.4%)	6 (5.9%)	2 (3.9%)	4 (8.0%)
More than one race	4 (5.0%)	1 (2.3%)	3 (8.1%)	4 (3.9%)	1 (1.9%)	3 (6.0%)
Unknown or not reported	2 (2.5%)	2 (4.7%)	0 (0%)	3 (2.9%)	2 (3.8%)	1 (2.0%)
**Annual family income**						
Less than USD 15,000	30 (37.5%)	15 (34.9%)	15 (40.5%)	34 (33.3%)	17 (32.7%)	17 (34.0%)
USD 15,000 to <29,999	23 (28.8%)	10 (23.3%)	13 (35.1%)	30 (29.4%)	13 (25.0%)	17 (34.0%)
USD 30,000 to <49,999	15 (18.8%)	10 (23.3%)	5 (13.5%)	22 (21.6%)	14 (26.9%)	8 (16.0%)
More than USD 50,000	10 (12.5%)	6 (14.0%)	4 (10.8%)	14 (13.7%)	7 (13.5%)	7 (14.0%)
Unknown or not reported	2 (2.5%)	2 (4.5%)	0 (0%)	2 (1.9%)	1 (1.9%)	1 (2.0%)
	**Cases with Complete** **SCL Data**			**Cases with Complete** **RSA Data**		
	**Overall** **(*n* = 46)**	**MCP** **(*n* = 29)**	**CP** **(*n* = 17)**	**Overall** **(*n* = 45)**	**MCP** **(*n* = 25)**	**CP** **(*n* = 20)**
	*m* (*sd*)	*m* (*sd*)	*m* (*sd*)	*m* (*sd*)	*m* (*sd*)	*m* (*sd*)
**Child’s age**	10.0 (0.49)	10.0 (0.54)	9.9 (0.43)	10.0 (0.45)	10.1 (0.49)	10.0 (0.39)
**Child’s reactive aggression 4th grade**	10.9 (2.26)	10.9 (2.25)	11.1 (2.33)	11.2 (2.54)	11.4 (2.41)	11.0 (2.74)
	*n* (%)	*n* (%)	*n* (%)	*n* (%)	*n* (%)	*n* (%)
**Child’s gender**						
Male	27 (58.7%)	18 (62.1%)	9 (52.9%)	28 (62.2%)	16 (64.0%)	12 (60.0%)
Female	19 (41.3%)	11 (37.9%)	8 (47.1%)	17 (37.8%)	9 (36.0%)	8 (40.0%)
**Child’s ethnicity**						
Hispanic or Latino	2 (4.3%)	1 (3.4%)	1 (5.9%)	2 (4.4%)	1 (4.0%)	1 (5.0%)
Not Hispanic or Latino	40 (87.0%)	26 (89.7%)	14 (82.3%)	41 (91.1%)	23 (92.0%)	18 (90.0%)
Unknown or not reported	4 (8.7%)	2 (6.8%)	2 (11.8%)	2 (4.4%)	1 (4.0%)	1 (5.0%)
**Child’s race**						
Black or African American	39 (84.8%)	25 (86.2%)	14 (82.4%)	37 (82.2%)	21 (84.0%)	16 (80.0%)
White or Caucasian	3 (6.5%)	2 (6.9%)	1 (5.9%)	4 (8.9%)	2 (8.0%)	2 (10.0%)
More than one race	2 (4.3%)	0 (0%)	2 (11.8%)	2 (4.4%)	0 (0%)	2 (10.0%)
Unknown or not reported	2 (4.4%)	2 (6.9%)	0 (0%)	2 (4.4%)	2 (8.0%)	0 (0%)
**Annual family income**						
Less than USD 15,000	16 (34.8%)	10 (34.5%)	6 (35.3%)	15 (33.3%)	7 (28.0%)	8 (40.0%)
USD 15,000 to <29,999	10 (21.7%)	5 (17.2%)	5 (29.4%)	12 (26.7%)	7 (28.0%)	5 (25.0%)
USD 30,000 to <49,999	12 (26.1%)	8 (27.6%)	4 (23.5%)	10 (22.2%)	6 (24.0%)	4 (2.0%)
More than USD 50,000	6 (13.0%)	4 (13.8%)	2 (11.8%)	5 (11.1%)	3 (12.0%)	2 (10.0%)
Unknown or not reported	2 (4.3%)	2 (6.9%)	0 (0%)	3 (6.7%)	2 (8.0%)	1 (5.0%)

MCP—Mindful Coping Power; CP—Coping Power; *n*—number of participants; *m*—mean; *sd*—standard deviation; USD—United States dollar; SCL—skin conductance level; RSA—respiratory sinus arrhythmia.

**Table 2:** Child behavioral outcomes at the 1-year follow-up.

	Coping Power (CP)					Mindful Coping Power (MCP)					
		Pre-Intervention		1-Year Follow-Up			Pre-Intervention		1-Year Follow-Up		ES
	*n*	Mean	S.D.	Mean	S.D.	*n*	Mean	S.D.	Mean	S.D.	
Social Skills	37	12.65	6.45	14.27	7.09	43	11.30	6.45	16.63	8.01	0.57
Externalizing Problems	37	26.86	17.75	33.59	21.47	43	29.81	20.95	29.37	21.32	−0.36
Reactive Aggression	36	9.19	4.04	9.75	4.26	42	9.71	3.68	8.50	4.01	−0.46
Proactive Aggression	36	7.08	3.11	6.13	2.70	42	7.38	3.47	5.57	2.97	−0.26

*n*—number of participants; S.D.—standard deviation; ES—effect size.

**Table 3:** MCP and CP effects on the teacher-rated child behavioral outcomes at the 1-year follow-up.

	**Social Skills (*n* = 80)**				**Externalizing (*n* = 80)**			
	***b***	***se***	***t*-Value**	***p***	***b***	***se***	***t*-Value**	***p***
Condition (MCP vs. CP)	−1.369	1.627	−0.84	0.402	1.974	4.526	0.44	0.664
Time	0.705	0.555	1.27	0.208	2.926	1.410	2.07	0.041
Time × condition	1.610	0.757	2.13	0.037	−3.118	1.924	−1.62	0.109
Time slope (CP)	0.705	0.555	1.27	0.208	2.926	1.410	2.07	0.041
Time slope (MCP)	2.316	0.515	4.50	<0.01	−0.192	1.308	−0.15	0.884
	**Reactive Aggression (*n* = 78)**				**Proactive Aggression (*n* = 78)**			
	***b***	***se***	***t*-value**	***p***	***b***	***se***	***t*-value**	***p***
Condition (MCP vs. CP)	0.278	0.885	0.31	0.753	0.154	0.687	0.22	0.823
Time	0.242	0.293	0.83	0.412	−0.411	0.261	−1.57	0.120
Time × condition	−0.770	0.399	−1.93	0.057	−0.376	0.356	−1.06	0.294
Time slope (CP)	0.242	0.293	0.83	0.412	−0.411	0.261	−1.57	0.120
Time slope (MCP)	−0.528	0.271	−1.95	0.055	−0.787	0.242	−3.25	0.002

MCP—Mindful Coping Power; CP—Coping Power; *b*—beta; *se*—standard error; *p*—*p*-value.

**Table 4:** Children’s autonomic nervous system effects at post-intervention.

	CP					MCP					ES
		Pre-Intervention		Post-Intervention			Pre-Intervention		Post-Intervention		
	*n*	Mean	S.D.	Mean	S.D.	*n*	Mean	S.D.	Mean	S.D.	
SCL Reactivity	17	3.20	3.10	0.76	1.28	29	2.06	3.10	1.69	2.69	0.66
RSA Reactivity	20	0.12	1.14	0.26	0.74	25	0.23	0.60	0.15	0.45	−0.25

CP—Coping Power; MCP—Mindful Coping Power; SCL—skin conductance level; RSA—respiratory sinus arrhythmia; ES—effect size.

**Table 5:** Intervention effects on the SCL and RSA reactivities.

	SCL Reactivity (*n* = 46)				RSA Reactivity (*n* = 45)			
	*b*	*se*	*t*-Value	*p*	*b*	*se*	*t*-Value	*p*
Condition (MCP vs. CP)	−1.322	0.792	−1.67	0.099	0.109	0.227	0.48	0.631
Time	−2.333	0.792	−2.95	0.005	0.166	0.222	0.75	0.459
Time × condition	2.205	0.964	2.29	0.027	−0.239	0.292	−0.82	0.418
Time slope (CP)	−2.333	0.792	−2.95	0.005	0.166	0.222	0.75	0.459
Time slope (MCP)	−0.128	0.596	−0.22	0.830	−0.073	0.192	−0.38	0.707

SCL—skin conductance level; RSA—respiratory sinus arrhythmia; MCP—Mindful Coping Power; CP—Coping Power; *b*—beta; *se*—standard error; *p*—*p*-value.

**Table 6:** Inhibitory control mediates reactive aggression outcomes at the one-year follow-up.

	Inhibitory Control (Post-Intervention)				Reactive Aggression (1-Year Follow-Up)			
	*b*	*se*	*t*-Value	*p*	*b*	*se*	*t*-Value	*p*
Condition (MCP vs. CP)	0.217	0.118	1.84	0.070	−1.382	0.752	−1.84	0.070
Inhibitory control (baseline)	0.329	0.082	4.00	<0.001	0.267	0.591	0.45	0.652
Inhibitory control (post-intervention)					−1.614	0.701	−2.30	0.024
Reactive aggression (baseline)	−0.047	0.015	−3.18	0.002	0.456	0.115	3.96	<0.001

MCP—Mindful Coping Power; CP—Coping Power; *b*—beta; *se*—standard error; *p*—*p*-value.

**Table 7:** Within-person associations with change in externalizing behavior problems.

	*b*	*se*	*t*-Value	*p*
RSA	6.605	2.183	3.03	<0.01
Reactive aggression	2.724	0.692	3.94	<0.01
Proactive aggression	0.507	0.836	0.61	0.549
Social skills	−0.183	0.290	−0.63	0.533
Time	3.394	1.146	2.96	<0.01

RSA—respiratory sinus arrhythmia; *b*—beta; *se*—standard error; *p*—*p*-value.

## Data Availability

The data presented in this study are available upon request from the corresponding author. The data will be made publicly available after all planned grant analyses have been published.
